# Bifunctional transcription factors: recent advances in growth and development, stress resistance, and quality formation of fruits and vegetables

**DOI:** 10.1093/hr/uhag100

**Published:** 2026-03-13

**Authors:** Pan Shu, Qinlu Zheng, Ziling You, Yun Zhu, Xi Cheng, Yuan Qing, Xin Yao, Jing Li, Lin Shen

**Affiliations:** Sichuan Technological Innovation Laboratory for South Subtropical Fruits, College of Agricultural Science, Xichang University, Xichang 615013, China; Sichuan Technological Innovation Laboratory for South Subtropical Fruits, College of Agricultural Science, Xichang University, Xichang 615013, China; Sichuan Technological Innovation Laboratory for South Subtropical Fruits, College of Agricultural Science, Xichang University, Xichang 615013, China; Sichuan Technological Innovation Laboratory for South Subtropical Fruits, College of Agricultural Science, Xichang University, Xichang 615013, China; Sichuan Technological Innovation Laboratory for South Subtropical Fruits, College of Agricultural Science, Xichang University, Xichang 615013, China; Sichuan Technological Innovation Laboratory for South Subtropical Fruits, College of Agricultural Science, Xichang University, Xichang 615013, China; Sichuan Technological Innovation Laboratory for South Subtropical Fruits, College of Agricultural Science, Xichang University, Xichang 615013, China; Sichuan Technological Innovation Laboratory for South Subtropical Fruits, College of Agricultural Science, Xichang University, Xichang 615013, China; College of Food Science and Nutritional Engineering, China Agricultural University, Beijing 100083, China

## Abstract

Fruits and vegetables are key components of the human diet, valued for their unique textures and flavors. In recent years, numerous studies have demonstrated that individual transcription factors (TFs) can simultaneously regulate two biological processes; these TFs are defined as bifunctional TFs. However, systematic reviews on these bifunctional TFs in fruits and vegetables remain limited. This review systematically summarizes current knowledge on bifunctional TFs in fruits and vegetables, focusing on three themes: (i) molecular mechanisms (cis-element diversity, partner switching, posttranslational); (ii) network topology (hubs vs bottlenecks); and (iii) agronomic trade-offs. Meanwhile, the functional conservation and divergence of homologous TFs in different fruits and vegetables have also been investigated. In addition, we elaborate how key TF families, including MYB, bHLH, WRKY, ERF, and NAC, regulate diverse physiological processes in fruits and vegetables via dual mechanisms. We also identify several limitations in the existing literature, such as insufficient understanding of bifunctional regulatory mechanisms, incomplete identification of target genes, and inadequate exploration of crop applications.

## Introduction

Fruits and vegetables provide essential vitamins, minerals, fiber, and bioactive compounds (such as phenolics and flavonoids). Their appealing flavor, color, and texture also enhance consumer enjoyment [1, 2]. In addition, improved yields and quality increase economic viability and market competitiveness [3]. Recent research has focused on elucidating the regulation of nutritional biosynthesis (e.g. sugars, acids, pigments, and vitamins), responses to abiotic (e.g. drought, salinity, temperature) and biotic (e.g. pathogens, pests) stresses, and organ development (such as fruit ripening, coloration, and morphogenesis) [4–6].

Previous studies have primarily investigated single genes or transcription factors (TFs) in isolated physiological processes. However, growing evidence indicates that certain TFs, defined as bifunctional, can simultaneously regulate two biological processes. For example, tomato SlMYB13 promotes phenolamide biosynthesis (by activating *BGC7*/*BGC11*) and drought tolerance (via enhanced ROS scavenging and ABA accumulation). Similarly, pepper CaNAC2c concurrently mediates heat tolerance and nematode immunity [7, 8]. These findings suggest promising strategies for multitrait crop enhancement. However, the current literature lacks systematic integration of three key aspects: (i) molecular mechanisms underlying bifunctional TF activity; (ii) species-specific investigations in fruit and vegetable crops; and (iii) key knowledge gaps and research bottlenecks. This review synthesizes the current understanding of bifunctional TFs in fruits and vegetables, with particular focus on three themes: (i) molecular mechanisms (cis-element diversity, partner switching, posttranslational); (ii) network topology (hubs vs bottlenecks); and (iii) agronomic trade-offs (Table S1). Moreover, we highlight critical limitations in existing research, including insufficient mechanistic insights, incomplete target gene identification, and limited studies on crop applications. These insights offer a foundation for molecular breeding strategies targeting multiple agronomic traits.

## Molecular mechanism of bifunctional TFs regulation

**Figure 1:**
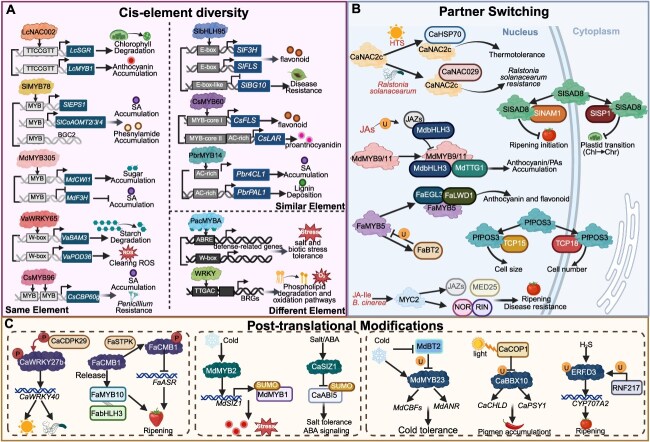
Regulatory mechanisms of bifunctional TFs in fruits and vegetables. (A) Cis-element diversity. Bifunctional TFs recognize distinct or identical cis-elements to activate or repress target gene expression. For example, LcNAC002 binds the TTCCGTT motif to regulate chlorophyll degradation and anthocyanin accumulation; SlbHLH95 interacts with E-box elements to modulate flavonoid biosynthesis and disease resistance; and PacMYBA employs ABRE, W-box, and TTAC motifs to coordinate defense responses and abiotic stress tolerance. (B) Partner switching. Interactions with different protein partners rewire the regulatory output of bifunctional TFs. CaNAC29 enhances thermotolerance when complexed with CaHSP70 but promotes resistance to *R. solanacearum* when paired with CaNAC2c. MdMYB9 and MdMYB11 interact with distinct bHLH proteins to regulate anthocyanin biosynthesis and stress responses, whereas FaMYB5 switches between FaEGL3 and FaBT2 to control fruit ripening and pigmentation. (C) Posttranslational modifications (PTMs). PTMs, including phosphorylation, SUMOylation, and ubiquitination, dynamically modulate the stability, localization, and activity of bifunctional TFs. Phosphorylation of CaWRKY27b releases CaWRKY40 to modulate disease resistance; SUMOylation of MdMYB1 affects stress signaling; ubiquitin-mediated degradation of CaBBX10 regulates light-induced pigment accumulation; and ubiquitination of ERF.D3 controls fruit ripening. Arrows indicate activation or positive regulation; lines with terminal bars indicate repression.

Bifunctional TFs can simultaneously regulate two biological processes via key molecular mechanisms, including cis-element diversity, partner switching, and posttranslational modifications (Fig. 1). Deciphering these molecular mechanisms not only reveals the intrinsic logic by which fruits and vegetables respond to complex environments while coordinating growth and development, but also provides theoretical foundations and design principles for targeted crop trait improvement.

### Cis-element diversity

Transcriptional regulation based on cis-element diversity is an important molecular basis enabling bifunctional TFs multifunctionality. These TFs can recognize and bind multiple cis-elements of the same type, multiple different types of cis-elements, or similar cis-elements within the same type, achieving differential regulation across target genes. For instance, in litchi, LcNAC002 directly binds to and activates the TTCCGTT cis-element in anthocyanin regulator *Linnaea chinensis MYELOBLASTOSIS1* (*LcMYB1*) and the key chlorophyll degradation gene *L. chinensis STAY-GREEN* (*LcSGR*). *LcSGR* promotes chlorophyll degradation and chloroplast disintegration by interacting with LcPAO. Meanwhile, LcMYB1 upregulates the expression of anthocyanin biosynthesis genes (e.g. *LcDFR* and *LcUFGT1*), integrating coloration process during fruit ripening [9]. In tomato, SlMYB78 directly regulates the BGC2 gene cluster (e.g. *SlEPS1*, *SlCoAOMT2/3/4*) by binding to the MYB cis-elements in their promoters. This promotes the phenylamide and salicylic acid (SA) biosynthesis, enhancing plant resistance to *Pst DC3000* [10]. In apples, MdMYB305 promotes sugar accumulation and reduces anthocyanin content by binding to the MYB cis-elements of sugar-related gene (e.g. *MdCWI1*, *MdVGT3*, and *MdTMT2*) and anthocyanin-related gene (e.g. *MdF3H*, *MdDFR*, and *MdUFGT*) promoters [11]. Similarly, low temperature induces grape *WRKY65* expression*,* which specifically binds to W-box elements in promoters of downstream target genes *VaBAM3* (starch degradation gene) and *VaPOD36* (antioxidant gene). This synergistically promotes starch breakdown and peroxidase activity, maintains osmotic balance and energy supply, clears reactive oxygen species (ROS), reduces oxidative damage, and enhances low-temperature adaptability [12]. To ensure robust and efficient regulation of key physiological processes, certain TFs adopt a ‘multisite binding’ reinforcement mechanism. In *Citrus, CsCBP60g* is the direct target of CsMYB96, and its promoter contains two natural isoforms, both harboring two MYB TF binding sites. CsMYB96 directly binds to and activates these MYB cis-elements, promoting the upregulation of *ICS1*, a key SA biosynthetic gene, and facilitating SA accumulation. Meanwhile, overexpression of *CsMYB96* increases total phenolic and flavonoid content, enhancing resistance to *Penicillium* [13].

In addition, some bifunctional TFs exert both activation and repression by binding to similar cis-elements. In tomato, SlbHLH95 binds to E-box motifs in the promoters of flavonoid biosynthesis genes *SlF3H* and *SlFLS*. It also interacts with similar E-box-like motifs in the promoter of the disease resistance gene *SlBG10*. This dual binding promotes flavonoid accumulation (e.g. rutin and nicotiflorin) in fruits and enhances disease resistance [14]. In cucumber, CsMYB60 coordinates the regulation of different branches within the flavonoid metabolic network by binding to similar MYB elements. Specifically, CsMYB60 promotes flavonol synthesis by binding to the type I MYB-core element (CAGTTG) in the *CsFLS* promoter, and enhances proanthocyanidin synthesis by binding to the type II MYB-core element (TGGTTG) and the AC-rich element (ACCCAC) in the *CsLAR* promoter [15]. In pear, PbrMYB14 similarly coordinates the enhancement of multiple defense pathways by recognizing similar cis-elements. Upon pathogen infection and exogenous SA treatment, *PbrMYB14* expression is induced. It directly binds to and activates similar cis-elements (e.g. AC-rich motifs) in the promoters of the key lignin synthesis gene *Pbr4CL1* and the SA synthesis gene *PbrPAL1*. This binding promotes lignin deposition, reinforcing the cell wall as a physical barrier, while simultaneously increasing SA accumulation to activate systemic defense signaling [16]. In sweet cherry, the promoter region of *PacMYBA* contains various stress-related cis-elements, including the ABA-responsive ABRE, SA-responsive TCA element, and JA-responsive TGACG-motif/CGTCA elements. Additionally, this region harbors pathogen defense-related elements such as W-box and Box-W1, which are binding sites for WRKY TFs. Upon induction, PacMYBA directly activates the expression of defense-related genes by specifically binding to cis-elements (e.g. ABRE and W-box) in the promoters of genes associated with salt stress tolerance and pathogen resistance, thereby synergistically enhancing plant tolerance to salt stress and biotic stress [17]. In the study of banana skin browning caused by low temperature, it was also found that WRKY TFs bind to the promoter of browning-related genes (*BRGs*) by recognizing diverse cis-elements (such as TTGAC motif), thereby activating phospholipid degradation and oxidation pathways [18]. These results confirm the important role of cis-element diversity in the regulatory mechanism of bifunctional TFs.

### Partner switching

Partner switching refers to the process by which a core signaling molecule (e.g. a transcription factor) sequentially binds to and dissociates from different downstream effector or regulatory proteins at various stages of a signaling pathway, thereby relaying the signal forward. Bifunctional TFs dynamically change their interacting protein partners according to cellular status, signal stimuli, or developmental stages, enabling precise regulation of physiological functions. In pepper, CaNAC2c employs a condition-dependent protein interaction mechanism to regulate heat tolerance and disease resistance in response to diverse signaling scenarios. Under high-temperature stress (HTS), CaNAC2c interacts specifically with CaHSP70 in the nucleus to protect CaNAC2c from degradation and enhance its binding to target promoters, thereby increasing transcriptional activation and improving thermotolerance. Conversely, upon infection by *Ralstonia solanacearum*, CaNAC2c switches interaction partners and forms a complex with CaNAC029 in the nucleus. Together, they activate jasmonic acid (JA)-mediated defense genes, such as *CaDEF1*, while simultaneously suppressing heat tolerance-related gene expression [8]. In apple, MdMYB9 and MdMYB11 form functional MBW complexes with MdbHLH3 and MdTTG1, directly activating anthocyanin and proanthocyanidin biosynthesis genes, thereby regulating the accumulation of anthocyanins and polyphenols. However, this process is strictly influenced by jasmonic acid (JA) signaling. Under normal conditions, JAZ inhibitory proteins (such as MdJAZ2) interact with MdbHLH3 to inhibit the formation of MBW complexes. When JA signaling occurs, JAZ protein is degraded through the ubiquitination pathway, releasing MdbHLH3, which rapidly changes its interaction state and binds to MdMYB9/MdMYB11 and MdTTG1 to drive complex assembly, thereby regulating the synthesis of anthocyanins and polyphenols [19]. JA signaling also plays an important role in regulating fruit ripening and disease resistance. Pathogen infection induces JA accumulation, prompting MYC2 to be released from the MYC2–JAZ complex and interact with the co-activator MED25, thereby activating the defense response. In addition, MYC2 interacts with mature regulatory factors NOR and RIN to form a MYC2–NOR/RIN protein complex, enhancing MYC2’s binding and transcriptional activation ability to the ERF.F4 promoter. They synergistically regulate fruit ripening and resistance to *Botrytis cinerea* [20]. Similarly, in strawberries, FaMYB5 dynamically regulates the biosynthesis of flavonoids by switching interaction partners based on internal metabolic demands. FaMYB5 forms a functional MYB–bHLH–WD40 (MBW) complex with the bHLH TF FaEGL3 and WD40 proteins, directly binding and activating key genes *F3′H* and *LAR* in the anthocyanin and flavonoid biosynthesis pathways to promote the accumulation of anthocyanins and flavonoids. In addition, FaMYB5 can switch its interaction partner to FaBT2, which promotes its own degradation and prevents excessive accumulation of metabolites [21]. In contrast, the partner-switching mechanism between the TF PfPOS3 and TCP protein partners (TCP15/TCP18) in *Physalis* appears primarily regulated by internal developmental programs. By forming specific heterodimers with distinct TCP partners (TCP15 or TCP18), PfPOS3 differentially regulates downstream cell-cycle genes. The POS3–TCP15 complex directly binds to and activates the transcription of CYCD1;1, promoting the G1-to-S phase transition. Conversely, the POS3–TCP18 complex directly binds to and represses the transcription of CYCB1;1, affecting the G2-to-M phase transition. This dual regulation coordinates the promotion of cell expansion (increasing cell size) and suppression of cell division (reducing cell number) during fruit development. Thus, it precisely controls organ size and weight [22]. SlSAD8 dually localizes to the nucleus and plastid to negatively regulate tomato fruit ripening via distinct protein interaction mechanisms. In the nucleus, SlSAD8 interacts with the ripening-associated TF SlNAM1, thereby repressing its transcriptional activation of ethylene biosynthesis genes and suppressing ethylene production and ripening initiation. In plastids, SlSAD8 associates with the E3 ligase SlSP1, stabilizing the chloroplast outer membrane protein Toc75 by inhibiting its ubiquitination and degradation. This leads to impaired chlorophyll synthesis and elevated chloroplast protein levels. Through this dual-compartment protein interaction network, SlSAD8 integrates organelle-specific signals to fine-tune the timing of fruit ripening and pigment metabolism [23].

### Posttranslational modifications

Beyond the previously discussed diversity of cis-elements and partner switching, bifunctional TFs are regulated by posttranslational modifications. These modifications influence their transcriptional activation and repression functions. Posttranslational modifications refer to covalent additions of chemical groups (e.g. phosphorylation, ubiquitination, or SUMOylation) or proteolytic cleavage processes occurring after protein synthesis. They enable rapid, reversible changes in protein activity, stability, or interactions. In pepper, CaWRKY27b positively regulates resistance to *R. solanacearum* (*RSI*) and tolerance to high-temperature and high-humidity (HTHH) stress through phosphorylation. Specifically, RSI infection or HTHH stress triggers Ca^2+^ influx, activating the calcium-dependent protein kinase CaCDPK29. Activated CaCDPK29 phosphorylates CaWRKY27b at Ser137, causing its translocation into the nucleus. The interaction between CaWRKY27b and CaWRKY40 enhances the ability of CaWRKY40 to bind to the W-box in promoters of target genes (e.g. *CaNPR1*, *CaDEF1*, and *CaHSP24*). This binding upregulates the expression of immunity- and thermotolerance-related genes [24]. During strawberry fruit ripening, phosphorylation also plays a crucial role. First, the MADS-box TF FaCMB1 competes with the core anthocyanin synthesis activator FaMYB10 for binding to FabHLH3, thereby preventing formation of the FaMYB10–FabHLH3 transcriptional complex and suppressing anthocyanin biosynthetic gene expression. Second, FaCMB1 directly binds to the promoter of the ABA-, stress-, and ripening-induced gene *FaASR* and represses its transcription. This repression relieves FaASR-mediated inhibition of the ABA metabolism gene *FaCYP707A4* (encoding ABA 8′-hydroxylase), leading to ABA accumulation and promoting fruit ripening. FaCMB1 activity is itself subject to phosphorylation by the kinase FaSTPK. Phosphorylation at Thr65 and Ser74 inhibits FaCMB1 binding to the *FaASR* promoter, thereby alleviating its transcriptional repression function [25].

In addition to phosphorylation, SUMOylation is another crucial posttranslational modification in the regulation of bifunctional TFs. As a key SUMO E3 ligase, SIZ1 plays a central role in abiotic stress responses. In apple, low-temperature signals induce *MdMYB2* expression, activating transcription of *MdSIZ1* by binding to the MBS cis-element in its promoter. Subsequently, MdSIZ1 SUMOylates the anthocyanin synthesis regulator MYB1, protecting it from degradation and promoting expression of downstream genes (*MdANS* and *MdDFR*). Thus, anthocyanin accumulation is enhanced. Furthermore, MdSIZ1-mediated SUMOylation stabilizes stress-responsive proteins, improving plant tolerance to cold, drought, and salt stress [26]. In pepper, another SUMO E3 ligase, CaSIZ1, is induced by salt stress or ABA treatment. This triggers SUMOylation modifications that regulate stress-responsive genes and enhance salt tolerance. Additionally, CaSIZ1 may regulate ABA signaling by directly binding and SUMOylating ABA signaling components such as *CaABI5* [27]. Ubiquitination is a highly conserved posttranslational modification regulating protein stability through mono- or polyubiquitination. Polyubiquitination typically targets proteins for degradation via the 26S proteasome, allowing precise control of cellular signaling pathways. In apple, low-temperature conditions induce the R2R3-MYB transcription factor MdMYB23, which directly binds MYB-recognition sites in promoters of *MdCBF1* and *MdCBF2*. This binding activates the CBF signaling pathway, enhancing cold tolerance. Additionally, MdMYB23 binds the promoter of the proanthocyanidin biosynthesis gene MdANR, promoting proanthocyanidin accumulation to scavenge ROS. This synergistically enhances cold adaptation. Crucially, the E3 ligase MdBT2 directly interacts with MdMYB23 via its TAZ domain, mediating its ubiquitination and subsequent degradation through the 26S proteasome pathway. However, low-temperature stress suppresses MdBT2 transcription, reducing MdMYB23 degradation. This stabilizes the protein and enhances its ability to activate downstream pathways [28]. Beyond anthocyanins, the biosynthesis and degradation of pigments such as chlorophyll and carotenoids are precisely regulated by ubiquitination. In pepper, the B-box TF CaBBX10 binds directly to the G-box cis-element in the promoters of chlorophyll biosynthesis gene *CaCHLD* and carotenoid biosynthesis gene *CaPSY1*. This binding activates their transcription, promoting coordinated accumulation of both pigments. However, the photomorphogenesis suppressor CaCOP1 interacts with CaBBX10 and mediates its ubiquitination and degradation through the 26S proteasome pathway, precisely controlling pigment accumulation under conditions like light exposure [29]. In tomato fruit ripening and disease resistance, ubiquitination also plays a significant role. Hydrogen sulfide (H_2_S) is a signaling molecule modulating plant senescence. The ethylene response factor ERF.D3 can be rapidly induced by H_2_S. Overexpression of ERF.D3 reduces abscisic acid (ABA) levels. Additionally, ERF.D3 enhances the transcriptional activity of CYP707A2, a key ABA catabolism enzyme gene, through persulfidation modification. Furthermore, the E3 ubiquitin ligase RNF217 ubiquitinates ERF.D3, potentially accelerating ripening in later fruit development stages. These findings indicate ERF.D3’s role in delaying leaf senescence and fruit ripening [30].

### The core regulatory role of bifunctional TFs in network topology

**Figure 2:**
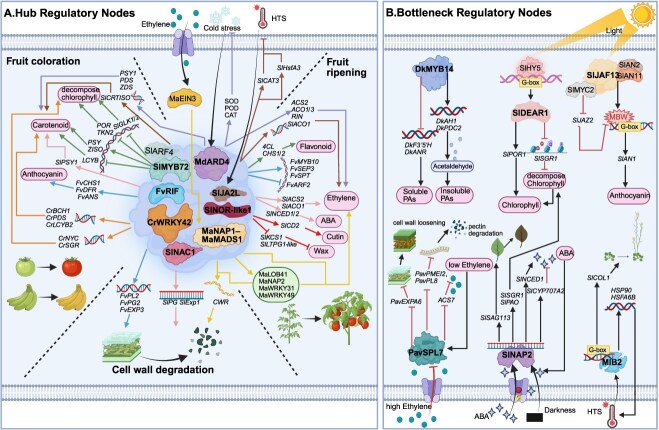
The core regulatory role of bifunctional TFs in network topology. (A) Hub regulatory nodes. Bifunctional TFs such as MdARD4, FvRIF, and MaNAP1/MaMADS1 function as central hubs that integrate signals from ethylene, cold stress, and heat stress (HTS) to regulate multiple downstream processes. These include fruit coloration (carotenoid and anthocyanin biosynthesis), fruit ripening (ethylene and ABA signaling), and cell wall degradation. By connecting diverse pathways, these nodes coordinate fruit maturation in species such as tomato, strawberry, and banana. (B) Bottleneck regulatory nodes. Bifunctional TFs including DkMYB14, SIDEAR1, and SINAP2 act as bottlenecks that mediate crosstalk among light, darkness, and HTS signals. They regulate proanthocyanidin (PA) accumulation, chlorophyll degradation, ethylene biosynthesis, and ABA signaling, thereby controlling fruit softening, ripening, and stress adaptation. These nodes serve as critical checkpoints that fine-tune developmental transitions and stress responses. Arrows indicate activation of gene expression or promotion of physiological processes. Arrow colors correspond to physiological pathways mediated by specific TFs. Lines with terminal bars indicate repression.

After examining how TFs achieve bifunctional plasticity through diverse molecular mechanisms, it is clear that regulatory features of certain TFs are not isolated. Instead, they often occupy central positions in complex regulatory networks. Many bifunctional TFs in fruits and vegetables, owing to their multitarget binding and integration of multiple pathways, serve as critical regulatory hubs or bottlenecks within network topology (Fig. 2).

### Bifunctional TFs as hub regulatory nodes

Bifunctional TFs act as hub regulatory nodes by linking multiple signaling pathways, integrating diverse upstream signals, and simultaneously controlling various downstream physiological processes. Thus, they exert broad effects on overall phenotype, specific developmental stages, or stress responses. In tomato, SlMYB72 interacts with the auxin response factor SlARF4, negatively regulating *SlGLK1* and *SlGLK2*, thus inhibiting chloroplast development. It also directly targets *PROTOCHLOROPHYLLIDE REDUCTASE* (*POR*), *Mg-CHELATASE H SUBUNIT* (*CHLH*), and *TKN2* genes to promote chlorophyll degradation. Simultaneously, SlMYB72 binds promoters of *PHYTOENE SYNTHASE* (*PSY*), *ζ-CAROTENE ISOMERASE* (*ZISO*), and *LCYB*, positively regulating *PSY* and *ZISO* but negatively regulating *LCYB* to enhance carotenoid synthesis. Additionally, it directly targets and downregulates flavonoid pathway genes *4-COUMARATE-COENZYME A LIGASE* (*4CL*), *CHALCONE SYNTHASE1* (*CHS1*), and *CHS2*, promoting flavonoid accumulation. Knocking out *SlMYB72* severely hinders fruit ripening and flavonoid synthesis, highlighting its hub position [31]. In strawberries, DAP-seq combined with transcriptome analysis identified 2080 genes as potential FvRIF-mediated targets. These genes, together with promoters of 137 TF genes (e.g. *FvMYB10* and *FvSEP3*), form a regulatory network. As a core regulator, FvRIF links the entire ripening process from color and texture to flavor. It directly binds promoters of anthocyanin biosynthesis genes (*FvCHS1*, *FvDFR*, *FvANS*), promoting fruit coloration. It also targets cell wall degradation genes (*FvPL2*, *FvPG2*, *FvEXP3*), regulating fruit softening. Simultaneously, FvRIF activates *FvMYB10*, *FvSEP3*, and *FvSPT*, while repressing *FvARF2*, thereby promoting flavor formation and fruit ripening [32]. However, SlNAC1 acts as a core regulatory node that integrates four distinct networks to inhibit fruit ripening and pigmentation. SlNAC1 binds promoters of key ethylene synthesis genes (*SlACS2*, *SlACO1*) and the carotenoid synthesis gene *SlPSY1*, repressing their expression. This reduces ethylene production and lycopene accumulation. Simultaneously, SlNAC1 upregulates ABA synthesis genes (*SlNCED1*, *SlNCED2*), enhancing ABA accumulation, and activates cell wall metabolism-related genes (*SlPG*, *SlExp1*), accelerating cell wall degradation and fruit softening [33]. Fruit and vegetable coloration is also closely linked to chlorophyll degradation and carotenoid accumulation. In *Citrus*, the transcription factor CrWRKY42 directly activates carotenoid biosynthesis genes (*CrBCH1*, CrPDS, *CrLCYB2*) and chlorophyll degradation genes (*CrNYC*, *CrSGR*), synchronously driving chlorophyll breakdown and carotenoid accumulation [34]. Bananas are among the world’s highest-yielding fruit crops but are prone to excessive softening and finger shedding, causing decay and pest infestation. The underlying mechanisms remain unclear. Recently, Li *et al.* [35] utilized multiomics approaches (RNA-seq, DNase-seq, ChIP-seq) to reveal that the MaNAP1–MaMADS1 cascade, activated by MaAPETALA3/PASTILLATA1 (MaNAP1) and MaMADS1, regulates 31 cell wall-related (CWR) genes and 713 secondary TFs (e.g. MaLOB41, MaNAP2, MaWRKY31, MaWRKY49). In addition, MaNAP1 and MaMADS1 are directly targeted by MaEIN3, enabling integration of MaEIN3-mediated ethylene signaling and epigenetic regulation [35]. These results demonstrate that the MaNAP1–MaMADS1-centered regulatory cascade promotes cell wall degradation and ethylene biosynthesis, regulating banana peel softening and finger shedding [35]. Such network topology, functioning as a multichannel integration hub, has been widely reported in traits related to fruit ripening and pigment accumulation. Recent studies indicate that many bifunctional TFs regulate temperature stress-mediated fruit ripening, fruit set, and fruit integrity. Acireductone dioxygenase (ARD) is a key enzyme in ethylene biosynthesis. Under cold stress, MdARD4 transcription is significantly upregulated. This enhances antioxidant enzyme activities (SOD, POD, CAT), reduces hydrogen peroxide and malondialdehyde accumulation, and improves membrane stability, thereby increasing cold tolerance. It also promotes fruit ripening by activating ethylene biosynthesis genes (*ACS2*, *ACO1*, *ACO3*, *RIN*) and upregulating carotenoid biosynthesis genes (*PSY1*, *PDS*, *ZDS*) [36]. The plant cuticle forms a critical protective barrier. In tomato, SlNOR-like1 finely controls fruit peel formation through two functional networks, cutin synthesis, and wax synthesis and transport. SlNOR-like1 activates cutin synthesis genes *SlGPAT6* and *SlCD2*, promoting cutin deposition. Conversely, it inhibits wax biosynthesis genes (*SlKCS1*, *SlCER1-2*, *SlWAX2*) and the wax transport gene SlLTPG1-like, limiting wax accumulation [37]. Certain bifunctional TFs function as hubs in network topology, coordinating fruit ripening, stress resistance, and associated signaling pathways. In thermotolerance regulation, SlJA2L enhances ROS scavenging by directly activating *SlCAT3*, while inducing heat shock protein (HSP)-encoding genes via activation of *SlHsfA3*, thereby improving plant heat tolerance. In ripening regulation, SlJA2L transcriptionally activates the ethylene biosynthesis gene *SlACO1* and the carotenoid metabolism enzyme gene *SlCRTISO*, synergistically promoting ethylene and abscisic acid (ABA) synthesis, accelerating fruit ripening and pigmentation, and thus establishing a bidirectional thermotolerance–ripening regulatory network in tomato [38]. Collectively, these studies demonstrate that bifunctional transcription factors act as hubs within network topology to coordinate ripening, quality, and stress resistance in fruits and vegetables.

### Bifunctional TFs as bottleneck regulatory nodes

Key bottleneck TFs in fruit and vegetable regulatory networks have rate-limiting or on–off control effects on specific signaling pathways or phenotypic traits. Soluble sugars and organic acids regulate fruit and vegetable flavor, while anthocyanins, amino acids, vitamins, flavonoids, and phenolics influence nutritional quality. Persimmons are popular due to their sweetness, unique aroma, and taste. However, the astringency of certain varieties limits marketability. Research indicates that persimmon transcription factor DkMYB14, acting as a regulatory switch, exerts dual effects: it inhibits proanthocyanidin (PA) synthesis by downregulating *DkF3′5′H* and *DkANR*, reducing astringency, and promotes acetaldehyde production by upregulating *DkAH1* and *DkPDC2*, increasing PA insolubility. These effects collectively control fruit astringency and enhance fruit quality [39]. Chlorophyll metabolism plays a crucial role in tomato fruit development and ripening. In immature fruits, chlorophyll supports photosynthesis, supplying growth energy. Conversely, chlorophyll degradation characterizes maturity in most tomato varieties. Under light conditions, the upstream transcription factor SlHY5 directly binds the G-box cis-element in the *SlDEAR1* promoter, activating its transcription. SlDEAR1, acting as a chlorophyll content regulator, directly binds the promoter of chlorophyll biosynthesis gene *SlPOR1*, promoting chlorophyll synthesis. Simultaneously, it reduces histone acetylation of the chlorophyll degradation gene *SlSGR1*, suppressing its transcription. This regulatory network maintains proper fruit coloration and ensures adequate photosynthetic energy supply in tomatoes [40]. Anthocyanins are natural plant pigments that play crucial roles in biological processes, including attracting pollinators and seed dispersers and protecting plants from biotic and abiotic stresses. In tomatoes, light activates the transcription factor SlJAF13, a key regulatory bottleneck. On one hand, SlJAF13 forms a ternary complex with SlAN2-like and SlAN11, binding to the promoter of SlAN1. This complex further assembles into an MBW complex, positively promoting anthocyanin synthesis. On the other hand, SlJAF13 interacts with SlMYC2, inhibiting the negative regulator SlJAZ2. This prevents SlJAZ2 from binding to the MBW complex, thus relieving its inhibitory effect on anthocyanin synthesis [41] (Fig. 2).

Ethylene treatment reduces transcription levels of PavSPL7, encoding a plant-specific SQUAMOSA promoter-binding protein-like (SPL) family member, accelerating cherry fruit softening. Overexpression of *PavSPL7* inhibits transcription of genes involved in cell wall loosening (*PavEXPA6*), pectin degradation (*PavPMEI2*, *PavPL8*), and ethylene synthesis (*ACS7*), thus suppressing fruit softening. These results indicate that ethylene and PavSPL7 antagonistically regulate cherry fruit softening through a negative feedback loop. During fruit development, low ethylene maintains high *PavSPL7* expression, inhibiting softening. As fruit matures, elevated ethylene downregulates *PavSPL7*, releasing its inhibition and finely controlling fruit softening [42]. In tomatoes, the NAC TF SlNAP2 functions as a central regulator of leaf senescence. *SlNAP2* expression gradually increases during leaf development, peaking in aging leaves. Upon activation by exogenous ABA and darkness, SlNAP2 directly binds to senescence marker gene *SlSAG113* and chlorophyll degradation genes *SlSGR1*/*SlPAO*, accelerating chlorophyll breakdown and leaf senescence. Additionally, SlNAP2 regulates ABA pathways by directly activating ABA biosynthesis gene *SlNCED1* and degradation gene *SlCYP707A2*, establishing a feedback loop to determine optimal senescence timing. Knocking down *SlNAP2* significantly delays leaf senescence and enhances photosynthesis, thus finely regulating fruit yield and sugar content [43]. HTS induces expression of the bHLH transcription factor MIB2 (SPATULA homolog), enhancing its binding to the G-box motif of the downstream gene *SlCOL1* promoter and activating transcription to promote inflorescence branching. MIB2 also activates heat shock protein genes (*HSP90*, *HSFA6B*), coordinating heat-stress protection and morphogenesis, thereby precisely regulating fruit size and yield [44]. These findings indicate that certain bifunctional TFs serve as critical regulatory hubs or bottlenecks within network topologies. They precisely regulate biological processes such as growth, development, quality formation, and stress responses in fruits and vegetables by mediating synergy or antagonism among various signaling pathways.

## Investigating the role of bifunctional TFs in agronomic trade-offs

**Figure 3:**
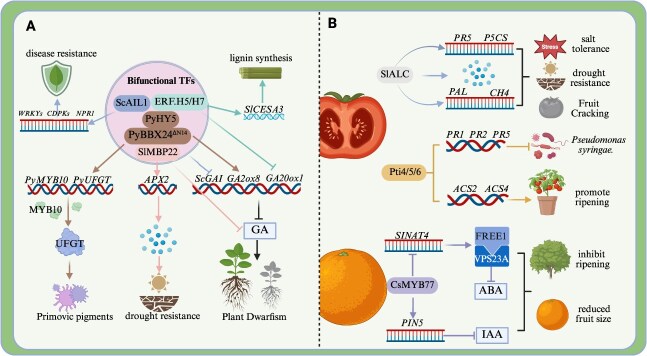
Bifunctional transcription factors mediate trade-offs in key agronomic traits of fruits and vegetables. (A) TFs (e.g. SlMBP22, PyBBX24^ΔN14^, ScAIL1, ERF.H5/H7) activate the expression of distinct target genes, leading to enhanced stress resistance or fruit development at the cost of growth trade-offs such as plant dwarfism. (B) TFs (e.g. SlALC, Pti4/5/6) primarily modulate plant responses to environmental stimuli and regulate processes associated with fruit quality. Arrows indicate activation, and lines with terminal bars indicate repression. Arrow colors correspond to specific transcription factor-mediated physiological pathways.

Traditional research has primarily examined individual traits, often overlooking interconnected phenotypic characteristics. This approach can result in undesirable trade-offs, such as increased stress resistance coupled with growth retardation, or high yield at the cost of fruit quality (Fig. 3). For example, the pear variant allele PyBBX24^ΔN14^ promotes anthocyanin biosynthesis by directly activating *PyUFGT* and *PyMYB10*, and through interaction with PyHY5. However, PyBBX24^ΔN14^ also directly binds to the *PyGA2ox8* promoter, upregulating its expression and consequently reducing endogenous gibberellin levels, causing plant dwarfism [45]. Similarly, tomato ERF.H5 and ERF.H7 enhance cell wall formation by activating cellulose biosynthesis gene *SlCESA3*, but simultaneously inhibit gibberellin biosynthesis gene *GA20ox1*, significantly reducing gibberellin (GA1, GA3, GA4) levels and leading to dwarfism [46]. *ABS (TT16)* is a MADS-box TF gene belonging to the Bsister (Bs) subfamily, which encodes proteins critical for plant development, regulating floral organogenesis, seed formation, and fruit growth. The tomato *SlMBP22* gene, an ABS homolog, is closely associated with plant morphology and abiotic stress responses. In growth regulation, *SlMBP22* induces plant dwarfing and increases chlorophyll and proline contents by modulating auxin (IAA) and gibberellin (GA) signaling pathways. Under drought stress, *SlMBP22* may participate in drought responses by regulating stress-related gene transcription, enabling active drought resistance [47]. In sugarcane, the AP2-family transcription factor ScAIL1 directly targets the *ScGAI* promoter, inhibiting GA biosynthesis and resulting in dwarfism. Concurrently, it activates defense-related genes (*WRKYs*, *CDPKs*, *NPR1*), enhancing pathogen resistance. These findings indicate inherent trade-offs between plant height, quality, and stress resistance [48]. In addition, trade-offs exist between fruit quality and resistance. SlALC encodes a bHLH family DNA-binding protein. Overexpression of *SlALC* promotes accumulation of osmotic regulators (proline, soluble sugars, starch) and activates antioxidant systems, enhancing drought and salt tolerance. However, it also downregulates lignin biosynthesis genes (*PAL*, *CH4*), reducing pericarp mechanical strength and increasing susceptibility to fruit cracking [49]. *Pseudomonas syringae* pv. tomato (*Pst*) causes tomato bacterial speck disease, significantly affecting tomato yield and quality. Overexpression of ERF TFs *Pti4/5/6* enhances pathogen resistance by upregulating *PR* gene expression*.* Yet, Pti4/5/6 also accelerates fruit ripening by activating ethylene synthesis genes (*ACS2*, *ACS4*) and the ripening-related regulator CNR [50]. In citrus, the transcription factor CsMYB77 binds directly to the *SINAT4* (*SEVEN IN ABSENTIA OF ARABIDOPSIS THALIANA4*) promoter, repressing its expression. Reduced *SINAT4* expression decreases degradation of FREE1/VPS23A proteins, enhances vacuolar degradation of ABA receptors (PYR1/PYL4), and weakens ABA signaling, thus delaying fruit ripening. Simultaneously, CsMYB77 directly activates *PIN-FORMED PROTEIN5* (*PIN5*), reducing free IAA content and resulting in smaller fruits [51]. These findings indicate that most identified TFs exhibit trade-offs between growth/development and resistance, as well as between quality and resistance. Therefore, future research should prioritize achieving simultaneous improvements in these traits (Fig. 3).

### Functional conservation and divergence of homologous TFs in different fruits and vegetables

**Figure 4:**
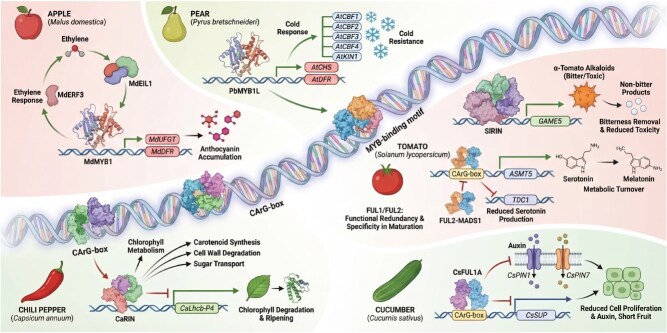
This schematic illustrates the functional roles of homologous TFs in five economically important fruit species: apple (*Malus domestica*), pear (*Pyrus bretschneideri*), tomato (*Solanum lycopersicum*), chili pepper (*Capsicum annuum*), and cucumber (*Cucumis sativus*).

MYB TFs play important roles in fruit and vegetable ripening, anthocyanin accumulation, and stress response. Homologous MYB TFs may exhibit functional conservation and divergence due to differences in their species origins. Ethylene signaling activated the expression of *MdEIL1* and promoted its direct binding and activation of MdMYB1, upregulating the expression of anthocyanin biosynthesis genes such as *MdDFR* and *MdUFGT*, thereby promoting anthocyanin accumulation and fruit coloring. Additionally, MdMYB1 activated the ethylene response factor 3 (MdERF3) to enhance ethylene accumulation, synergistically increasing anthocyanin accumulation and accelerating the fruit ripening process [52]. These results demonstrated that MdMYB1 played a positive role in anthocyanin synthesis and ethylene response in apples. A homologous MYB1 (PbMYB1L) was also found in pear fruit, which can promote anthocyanin accumulation by upregulating anthocyanin synthesis structural genes *AtCHS*, *AtCHI*, and *AtDFR*. Interestingly, PbMYB1L also acts as a positive regulatory factor to activate the CBF cold response pathway, significantly upregulating the expression of *AtCBF1*, *AtCBF2*, *AtCBF3*, *AtCBF4*, and downstream genes *AtKIN1* in *Arabidopsis*, enhancing antioxidant capacity and improving cold resistance [53]. During the ripening process of chili fruit, the expression of *CaRIN* is upregulated. It also binds to the promoter of the gene encoding chlorophyll a/b binding protein P4 (CaLhcb-P4) to inhibit its expression, thereby actively regulating chlorophyll degradation. Meanwhile, CaRIN indirectly regulates the expression of various genes such as chlorophyll metabolism, carotenoid synthesis, cell wall degradation, and sugar transport [54]. In tomatoes, although SlRIN and CaRIN belong to the MADS box transcription factor family and regulate the physiological process of fruit ripening, their functions are not the same. The SlRIN specifically binds to two CArG box motifs in the promoter of *GAME5*, thereby activating the transcription of GAME5. The increase of GAME5 protein can convert bitter and toxic sugar alkali intermediates (such as α-tomato alkaloids) into nonbitter and low-toxicity product. This regulatory process is crucial for removing bitterness and reducing food toxicity in the late stage of fruit ripening [55]. During tomato fruit development and ripening, the TFs FUL2 and MADS1 form functional heterodimers that directly bind to CArG-box motifs in the promoter of *ASMT5* and activate its transcription. ASMT5 converts serotonin to melatonin via acetylation, thereby promoting serotonin metabolic turnover. In addition, the FUL2–MADS1 heterodimer represses TDC1, which encodes a rate-limiting enzyme in serotonin biosynthesis, thus reducing serotonin production. Through this dual mechanism of enhancing metabolism while suppressing synthesis, the heterodimer effectively prevents excessive serotonin accumulation in fruit. Tomato has a functionally similar FUL2 homolog, FUL1. Loss of either FUL1 or FUL2 alone does not affect normal phenotypes, but the double mutant severely impairs ripening. Nonetheless, their functions have diverged: FUL2 mutation reduces fruit size, whereas FUL1 mutation does not [56]. In cucumber, CsFUL1 is an ortholog of tomato SlFUL2, and their functions have also diverged. CsFUL1^A^ is a gain-of-function allele of CsFUL1 and is specifically found in East Asian long-fruited cucumber accessions. The CsFUL1^A^ protein binds to CArG-box sites in the CsSUP gene, repressing its expression while simultaneously suppressing transcription of the auxin transporter genes *CsPIN1* and *CsPIN7*. This synergistic effect inhibits pericarp cell proliferation and expansion, reduces auxin accumulation, and ultimately negatively regulates fruit length [57]. These results confirm the conservation and differentiation of homologous genes in terms of function (Fig. 4). Although the pathways and physiological functions regulated by these transcription factors have different emphases, they still retain their core functions. Exploring the functional evolution of these TFs is crucial for transferring knowledge from the pattern system to other crops (Fig. 4).

## Conclusion and prospect

To meet the growing global demand for food, improving both crop yield and quality has become equally critical in agricultural research. Fruits and vegetables, rich in health-promoting compounds such as vitamins, flavonoids, and polyphenols, are essential components of the human diet. While some dual-function TFs have been confirmed in fruits and vegetables, future research still needs to be deepened and expanded across multiple dimensions. For instance, SlDQD/SlSDH2 has been shown to be transcriptionally regulated by the MADS-box protein SlTAGL1 [58]. Nevertheless, the potential molecular interactions among SlDQD/SDH2, SlMYB12, and SlTAGL1 in modulating flavonoid accumulation remain poorly understood. Moreover, current studies often concentrate on individual TFs, overlooking the collaborative effect of members within the same family or interacting partners. While both CaWRKY40 and CaNAC2c enhanced disease resistance in pepper, their concurrent overexpression could lead to hyperactivation of JA signaling, disrupting the balance between defense and development [8, 24]. In addition, most investigations rely on single-stress treatments, which fail to mimic field conditions involving combined stresses. For example, the CaWRKY40–JA/SA pathway is often validated using single-pathogen inoculations (e.g. *R. solanacearum*), neglecting dynamic JA/SA ratio changes under HTS. Under combinatorial stress, plants may face a trade-off between resistance and sensitivity. SlSRN1 positively regulates resistance to *B. cinerea* infection but suppresses drought tolerance [59]. Furthermore, while many TFs promote ripening through ethylene induction, elevated ethylene levels accelerate fruit softening and reduce shelf life. Identifying strategies that enhance stress resistance without compromising quality is crucial for advancing fruit and vegetable crop production.

To bridge fundamental discoveries with practical breeding applications, several translational strategies should be emphasized in future research. First, mining superior allelic variants of key TFs from diverse germplasm resources could provide valuable genetic materials for breeding programs. Natural variations in TF sequences often underlie differences in stress tolerance, metabolite accumulation, or developmental timing, and their identification would enable marker-assisted selection for desirable traits. Second, applying gene-editing technologies such as CRISPR-Cas9 offers a precise means to modify TF functions. It is possible to enhance beneficial traits while avoiding the negative effects of pleiotropy by knocking out or suppressing those unfavorable transcription factor alleles that impair stress resistance or quality traits, or by fine-tuning the expression of positive regulators. Third, designing pyramid breeding strategies based on collaborative TF regulatory networks facilitates the integration of multiple favorable traits. By stacking alleles of complementary TFs that co-coordinate growth, defense, and mass formation, it is possible to achieve balanced crop performance under complex environmental conditions. Harnessing bifunctional TFs, together with these applied approaches, may pave the way for developing crops with multistress resilience, balanced growth-defense trade-offs, concurrent accumulation of nutritional compounds, and synchronized improvement of quality and yield stability.

## Supplementary Material

Web_Material_uhag100
