# Supplementary material for: Bifunctional transcription factors: recent advances in growth and development, stress resistance, and quality formation of fruits and vegetables
Source: Hortic Res. 2026 Mar 13;13(7):uhag100. doi: 10.1093/hr/uhag100 (PMC13282542; doi:10.1093/hr/uhag100)
Supplement: Web_Material_uhag100 [file web_material_uhag100.docx]

| Table S1 Summary of results for bifunctional transcription factors | | | | |
| --- | --- | --- | --- | --- |
| **Themes** | **Sub-themes** | **TFs** | **Species** | **Reference** |
| Molecular Mechanism of Bifunctional TFs Regulation | Cis-element Diversity | LcNAC002 | *Litchi chinensis* | Zou et al. (2023) |
|  |  | SlbHLH95 | *Solanum lycopersicum* | Su et al. (2025) |
|  |  | SlMYB78 |  | Cao et al. (2025) |
|  |  | MdMYB305 | *Malus domestica* | Zhang et al. (2023) |
|  |  | VaWRKY65 | *Vitis vinifera* | Meng et al. (2025) |
|  |  | CsMYB96 | *Citrus reticulata* | Zhang et al. (2021) |
|  |  | CsMYB60 | *Cucumis sativus* | Li et al. (2020) |
|  |  | PbrMYB14 | *Pyrus spp* | Yan et al. (2025) |
|  |  | PacMYBA | *Prunus avium* | Guo et al. (2017) |
|  | Partner Switching | NAC2c | *Capsicum annuum* | Cai et al. (2021) |
|  |  | MdMYB9 and MdMYB11 | *Malus domestica* | An et al. (2015) |
|  |  | MYC2 | *Solanum lycopersicum* | Li et al. (2025) |
|  |  | SlSAD8 |  | Xu et al. (2025) |
|  |  | FaMYB5 | *Fragaria × ananassa* | Jiang et al. (2023) |
|  |  | PfPOS3 | *Physalis floridana* | Li et al. (2025) |
|  | Post-translational Modifications | CaWRKY27b | *Capsicum annuum* | Li et al. (2021) |
|  |  | CaSlZ1 |  | Lei et al. (2022) |
|  |  | CaBBX10 |  | Wang et al. (2025) |
|  |  | FacMB1 | *Fragaria × ananassa* | Jia et al. (2025) |
|  |  | MdMYB2 | *Malus domestica* | Jiang et al. (2022) |
|  |  | MdMYB23 |  | An et al. (2018) |
|  |  | ERF.D3 | *Solanum lycopersicum* | Hu et al. (2024) |
| The Core Regulatory Role of Bifunctional TFs in Network Topology | Bifunctional TFs as Hub Regulatory Nodes | SlMYB72 | *Solanum lycopersicum* | Wu et al. (2020) |
|  |  | SlNAC1 |  | Ma et al. (2014) |
|  |  | SlNOR-like1 |  | Liu et al. (2024) |
|  |  | SlJA2L |  | Liang et al. (2025) |
|  |  | MdARD4 |  | Guo et al. (2020) |
|  |  | FvRIF | *Fragaria vesca* | Li et al. (2023) |
|  |  | CrWRKY42 | *Citrus reticulata* | Chen et al. (2024) |
|  |  | MaNAP1 and MaMADS1 | *Musa spp* | Li et al. (2025) |
|  | Bifunctional TFs as Bottleneck Regulatory Nodes | DkMYB14 | *Diospyros kaki* | Hen et al. (2021) |
|  |  | SlDEAR1 | *Solanum lycopersicum* | Pei et al. (2025) |
|  |  | SlJAF13 |  | Chen et al. (2022) |
|  |  | SlNAP2 |  | Ma et al. (2018) |
|  |  | MIB2 |  | Sun et al. (2024) |
|  |  | PavSPL7 | *Prunus avium* | Sun et al. (2026) |
| Investigating the Role of Bifunctional TFs in Agronomic Trade-offs | | PyBBX24 | *Pyrus spp* | Yang et al. (2024) |
|  |  | SlERF.H5 and SlERF.H7 | *Solanum lycopersicum* | Pei et al. (2025) |
|  |  | SlMBP22 |  | Li et al. (2020) |
|  |  | SlALC |  | Gao et al. (2024) |
|  |  | Pti4/5/6 |  | Wang et al. (2021) |
|  |  | ScAIL1 | *Saccharum officinarum* | Chai et al. (2022) |
|  |  | CsMYB77 | *Citrus reticulata* | Zhang et al. (2024) |
| Functional Conservation and Divergence of Homologous TFs in Different Fruits and Vegetables | | MdMYB1 | *Malus domestica* | An et al. (2018) |
|  |  | PbMYB1L | *Pyrus bretschneideri* | Zhou et al. (2024) |
|  |  | CaRIN | *Capsicum annuum* | Song et al. (2025) |
|  |  | SlRIN | *Solanum lycopersicum* | Xie et al. (2024) |
|  |  | FUL2 |  | Xu et al. (2026) |
|  |  | CsFUL1 | *Cucumis sativus* | Zhao et al. (2019) |
